# O044: Provision of alcohol-based handrub products to WHO regions in 2011

**DOI:** 10.1186/2047-2994-2-S1-O44

**Published:** 2013-06-20

**Authors:** D Pittet, C Kilpatrick, A Belloli, J Storr, B Allegranzi, E Kelley

**Affiliations:** 1 University Hospitals Geneva, Geneva, Switzerland; 2 World Health Organisation, 1211 Geneva, Switzerland; 3 Imperial College London, London, UK

## Introduction

A collaboration between WHO Patient Safety and industry was established in 2012; Private Organisations for Patient Safety (POPS). One formal POPS project was undertaken at the end of 2012 to meet the aim of addressing system change, as part of a multimodal strategy to reduce health care-associated infections; a survey on the provision of alcohol-based handrub (ABHR), as this has been proven to increase compliance with hand hygiene and improve patient outcomes.

## Objectives

By undertaking surveys, collate and describe information on global and regional ABHR sales in the year 2011.

To provide recommendations on addressing the gaps in availability.

## Methods

In Nov ‘12 a survey in the form of a MS Excel spreadsheet sent to POPS participants, asked for volume of ABHR liters sold to healthcare by country and region. Basic analysis allowed for collation of total sales volumes by country; mapping these to the official WHO regions to allow for regional totals. Quality checks were undertaken by sharing final totals with participants and data were anonymised. Median and inter-quartile ranges for country sales were calculated using MS Excel.

## Results

In 2011, the global total of ABHR healthcare sales was 41,827,389 liters. The sales in country ranged from 0 to 16,076,612. Totals by WHO region were Africa 245,585; Americas 15,246,296; Eastern Mediterranean 747,285; Europe 32,849,769; South East Asia 100,794; Western Pacific 2,288,300. Four ranges of countries have been presented against a world map.

## Conclusion

A number of limitations exist including not all global distributors of ABHR being involved in the survey, the time period covering only a single year, no true denominator to base the numerator of healthcare sales being known, and that healthcare delivery varies between countries. This information has however provided WHO with key intelligence on the gaps in availability of a life-saving technology that is contributing to the global burden of health care-associated infections. Zero sales could mean a data gap, delivery gap or an unknown factor, however these results provide a solid starting point for the development of POPS project proposals to ensure affordable, reliable supplies of ABHR in all countries of the world to support patient safety.

## Disclosure of interest

None declared.

